# Antifungal Effect of Novel 2-Bromo-2-Chloro-2-(4-Chlorophenylsulfonyl)-1-Phenylethanone against *Candida* Strains

**DOI:** 10.3389/fmicb.2016.01309

**Published:** 2016-08-25

**Authors:** Monika Staniszewska, Małgorzata Bondaryk, Magdalena Wieczorek, Eine Estrada-Mata, Héctor M. Mora-Montes, Zbigniew Ochal

**Affiliations:** ^1^National Institute of Public Health-National Institute of HygieneWarsaw, Poland; ^2^Departamento de Biología, División de Ciencias Naturales y Exactas, Universidad de GuanajuatoGuanajuato, Mexico; ^3^Faculty of Chemistry, Warsaw University of TechnologyWarsaw, Poland

**Keywords:** sulfone, *Candida*, virulence factors, resistance, paradoxical growth

## Abstract

We investigated the antifungal activity of novel a 2-bromo-2-chloro-2-(4-chlorophenylsulfonyl)-1-phenylethanone (compound **4**). The synthesis of compound **4** was commenced from sodium 4-chlorobenzene sulfinate and the final product was obtained by treatment of α-chloro-β-keto-sulfone with sodium hypobromite. The sensitivity of 63 clinical isolates belonging to the most relevant *Candida* species toward compound **4** using the method M27-A3 was evaluated. We observed among most of the clinical strains of *C. albicans* MIC ranging from 0.00195 to 0.0078 μg/mL. Compound **4** at 32 μg/mL exhibited fungicidal activity against nine *Candida* strains tested using the MFC assay. Compound **4** displayed anti-*Candida* activity (with clear endpoint) against 22% of clinical strains of *Candida*. Under compound **4**, *Candida* susceptibility and tolerance, namely paradoxical effect (PG), was found for only two clinical isolates (*C. glabrata* and *C. parapsilosis*) and reference strain 14053 using both M27-A3 and MFC method. We found that compound **4** does not induce toxicity *in vivo* against larvae of *Galleria mellonella* (≥97% survival) and it displays reduced toxicity on mammalian cells *in vitro* (< CC_20_ at 64 μg/mL). Furthermore, XTT assay denoted clear metabolic activity of sessile cells in the presence of compound **4**. Thus, the effect of compound **4** on formed *C. albicans* biofilms was minimal. Moreover, strain 90028 exhibited no defects in hyphal growth on Caco-2 monolayer under compound **4** influence at MIC = 16 μg/mL. The MIC values of compound **4** against *C. albicans* 90028, in medium with sorbitol did not suggest that compound **4** acts by inhibiting fungal cell wall synthesis. Our findings with compound **4** suggest a general strategy for antifungal agent development that might be useful in limiting the emergence of resistance in *Candida* strains.

## Introduction

Members of the *Candida* genus are the third most common cause of hospital-acquired systemic infections, which are associated to high mortality rates worldwide (Falagas et al., 2010). Five major classes of antifungal drugs (fluoropyrimidine analogs, polyenes, azoles, allylamines, and echinocandins) are currently available and target different metabolic pathways, namely cell wall synthesis, pyrimidine salvage, or act by binding to ergosterol and cause osmotic leakage. Although they are effective in the treatment of candidiasis, the main disadvantages of these antimycotics, are the high toxicity to host tissues, emergence of drug-resistant strains, and high costs (Rajeshkumar and Sundararaman, 2012). In light of this, it has become essential to develop new drugs designed to circumvent resistance or target alternative cellular processes. Our early reports on the antifungal potential of the newly synthesized halogenated methyl sulfones (Staniszewska et al., 2015a,b) has spurred significant interest in the synthesis of new α-chloro β-keto-based sulfone which may offer improved antifungal activity.

It has been reported that paradoxical growth could increase the probability of the appearance of stable resistance among fungal strains and treatment failure (Rueda et al., 2014). This phenomenon was described mainly to echinocandins and fluconazole at high concentrations (Chamilos et al., 2007; Zanette and Kontoyiannis, 2013). As its mechanisms are incompletely understood, further studies on the mechanism of the newly synthesized agents paradoxical effect in regard to different *Candida* species emerged from studies of other authors (Chamilos et al., 2007; Zanette and Kontoyiannis, 2013; Juvvadi et al., 2015).

The ability of *C. albicans* to form biofilms is a major virulence attribute and a challenge to be managed (Maiolo et al., 2014; Gulati and Nobile, 2016). The effect of biofilm formation on pathogenicity and their inherent resistance to the majority of known antifungals emphasize the need for the development of new antifungals with efficiency against the biofilm mode of growth (Gulati and Nobile, 2016; Martins et al., 2016; Rajendran et al., 2016). Furthermore, biofilm formation has been reported as an independent predictor of mortality, in addition to inadequate antifungal therapy (Rajendran et al., 2016). *Candida albicans* is polymorphic fungus with the ability to form yeast and hyphal cells, both critical for biofilm development and maintenance (Gulati and Nobile, 2016). Our recent studies (Bondaryk et al., 2015; Staniszewska et al., 2015a,b) demonstrated that inhibition of hyphal growth attenuated virulence in an *in vitro* model of superficial candidiasis, and a disruption of this cellular process might serve as an effective therapeutic intervention. Thus, inhibition of dimorphism may provide an alternative approach allowing finding compounds with the potential to control *C. albicans* infections (Lu et al., 2014). Hyphae are required for stable biofilm formation, which suggests that this cell type has a unique role in this process (Finkel and Mitchell, 2011).

Here we identify and characterize the novel β-ketosulfone derivative 2-bromo-2-chloro-2-(4-chlorophenylsulfonyl)-1-phenylethanone, the first to our knowledge active against *C. albicans* wild-type reference strains and a large number of clinical isolates. We sought to determine whether clinical isolates are able to grow at β-ketosulfone concentrations higher than the MIC, which is termed the Paradoxical Growth effect (PG). β-ketosulfone was tested for its ability to destroy pre-formed *C. albicans* biofilm. We investigated β-ketosulfone mechanism of action, including the effects of the compound on the cell wall, cell morphology, and viability, as well as the yeast-to-hyphal form transition. We estimated the cytotoxic effect of β-ketosulfone against mammalian cells *in vitro* and *Galleria mellonella in vivo*.

## Materials and methods

### Synthesis of 2-bromo-2-chloro-2-(4-chlorophenylsulfonyl)-1-phenylethanone (compound 4)

**Figure 1:**
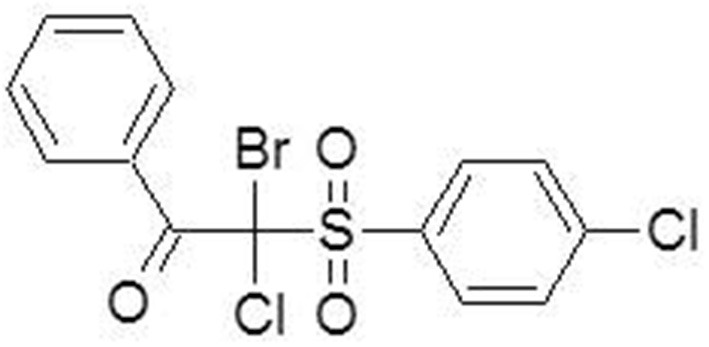
**Chemical structure of β-ketosulfone (compound 4)**.

The synthesis of 2-bromo-2-chloro-2-(4-chlorophenylsulfonyl)-1-phenylethanone (compound **4**, Figure 1) was commenced from sodium 4-chlorobenzene sulfinate. The final product was obtained by treatment of α-chloro-β-keto-sulfone with sodium hypobromite (Staniszewska et al., 2014 Polish Patent Application PL-P.408765). Detail procedures and characteristic data of synthesized compounds are given below.

### Synthesis of 2-(4-chlorophenylsulfonyl)-1-phenylethanone (2)

All reactions were set up in the air and using undistilled solvents. The reagents were purchased from commercial sources and were used without further purification. The reported yields refer to pure isolated products. Reactions were monitored by gas chromatography (GC) and/or thin layer chromatography (TLC) carried out on silica gel plates, using UV light. 1 H NMR spectra were recorded on a Varian Mercury 400 MHz spectrometer in CDCl3, using TMS (tetramethylsilane) as an internal standard; all signals are reported in ppm as (s = singlet, m = multiplet, integration). Mass spectra were recorded with a Micro-mass ESI Q-TOF spectrometer. All melting points (m.p.) are given uncorrected.

Compound (**2**) was synthesized according to the procedure described in publication (Suryakiran et al., 2006). A mixture of the sodium 4-chlorobenzene sulfinate (2.2 g, 11 mmol) and the 2-bromo-1-phenylethanone (2 g, 10 mmol) was taken in 50 mL of polyethylene glycol (PEG 400), and stirred at room temperature for 2 h. After completion of the reaction, as monitored by TLC, the reaction mass was poured into water (100 mL) and extracted into ethyl acetate (3 • 20 mL). The organic layer was removed under reduced pressure, and the crude product was purified by crystallization from ethanol. Yield 86%, m.p. 134–135 oC.1H NMR (400 MHz, CDCl3) δ 4.53 (s, 2H), 7.58–7.93 (m, 9H). 13C NMR (100 MHz, CDCl3) δ 171.96, 141.47, 137.8, 136.31, 135.62, 132.97, 129.84, 129.65, 127.71, 64.65.

### 2-chloro-2-(4-chlorophenylsulfonyl)-1-phenylethanone (3)

Compound (**3**) was synthesized in analogy to the procedure described in the paper (Suryakiran et al., 2007). To the solution of 2-(4-chlorophenylsulfonyl)-1-phenylethanone (2.95 g, 10 mmol) in acetic acid (10 mL) was added KCl (0.82 g 11 mmol) and 30% hydrogen peroxide (80 mmol). The reaction was stirred at room temperature for 10 h. After completion of the reaction, as monitored by TLC, the acetic acid was removed under reduced pressure, water (10 mL) was added and the product extracted into ethyl acetate (3 • 20 mL). The combined organic extracts were dried over anhydrous sodium sulfate and evaporated under reduced pressure to give the crude product, which was purified on a silica gel column using hexane: ethyl acetate (9:2) as eluent. Yield 76%, m.p. 145.5–146.5°C. 1H NMR (400 MHz, CDCl3) δ 6.24 (s, 1H), 7.65–8.64 (m, 9H). 13C NMR (100 MHz, CDCl3) δ 171.96, 144.77, 137.4, 135.88, 133.76, 132.36, 130.91, 130.48, 128.23, 69.35.

### 2-bromo-2-chloro-2-(4-chlorophenylsulfonyl)-1-phenylethanone (4)

2-Chloro-2-(4-chlorophenylsulfonyl)-1-phenylethanone (**3**) (3.19 g, 10 mmol) was added to freshly prepared 14% w/w aq sodium hypobromite (80 mL), and the resulting mixture was stirred at 75°C for 4 h. After cooling the mixture to ambient temperature, the precipitate was filtered off, washed with water, dried, and recrystallized from 2-propanol. Product (**4**) was obtained in 86% yield (3.55 g, 8.70 mmol); mp 165–166°C. 1H NMR (400 MHz, CDCl3) δ 7.63–8.52 (m, 9H).). 13C NMR (100 MHz, CDCl3) δ 172.93, 146.27, 137.38, 136.71, 135.62, 132.48, 130.95, 129.84, 129.65, 69.52. HRMS (ESI+, m/z): [M+H]+, found 406.8874. C14H9BrCl2O3S, requires 406.8905.

### Strains, media, and growth conditions

The wild type reference strains and the clinical isolates used in the study are listed in Table 1. The clinical isolates were acquired from Maria Skłodowska—Curie Oncology Centre, Hospital of Warsaw Medical University and Hospital of Infectious Diseases in Warsaw, and used throughout the study. *Candida* isolates obtained from both sterile and non-sterile sites were collected from 2012 to 2014. The isolates were identified based on colony color on CHROMagar *Candida* medium and evaluated following API 20C AUX carbohydrate assimilation patterns (Madhavan et al., 2011). Strains were stored on ceramic beads in a Microbank tube (Prolab Diagnostics, Canada) at −70°C. Prior to the respective examinations, routine cultures were conducted at 30°C for 18 h in Yeast extract-peptone-dextrose medium (YEPD; Staniszewska et al., 2015b). The study was approved by the Ethics Committee of the National Institute of Public Health-National Institute of Hygiene (Opinion 5/2012, 28.06.2012 and Appendix 18.04.2013) and all patients signed an informed consent form.

**Table 1:** **Strains used in current study**.

**Organism**	**Strain designation and sample number**	**Origin**
*Candida albicans (C.a.)*	90028 ATCC	Reference
	14053 ATCC	Reference
	28836 ATCC	Reference
	C.a. 82	Blood sample
	C.a. 129	Blood sample
	C.a. 171	Blood sample
	C.a. 87	Drain swab
	C.a. 96	Nose swab
	C.a. 97	Nose swab
	C.a. 128	Sputum sample
	C.a. 131	Stool sample
	C.a. 15	Vaginal swab
	C.a. 16	Vaginal swab
	C.a. 18	Vaginal swab
	C.a. 28	Vaginal swab
	C.a. 5	Wound swab
	C.a. 86	Wound swab
	C.a. 89	Wound swab
*Candida dubliniensis (C.d.)*	MYA-581 ATCC	Reference
*Candida glabrata (C.g.)*	C.g. 118	Anus swab
	C.g. 144	Aspirate sample
	C.g. 44	Blood sample
	C.g. 4	Drain swab
	C.g. 40	Drain swab
	C.g. 70	Drain swab
	C.g. 85	Drain swab
	C.g. 88	Drain swab
	C.g. 92	Drain swab
	C.g. 99	Drain swab
	C.g. 67	Nose swab
	C.g. 69	Nose swab
	C.g. 117	Oral cavity swab
	C.g. 41	Stoma swab
	C.g. 73	Wound swab
	C.g. 74	Wound swab
	C.g. 75	Wound swab
*Candida kefyr (C.k.)*	C.k. 27	Vaginal swab
*C. krusei (C.kr.)*	6258 ATCC	Reference
	C.kr. 122	Aspirate sample
	C.kr. 119	Bile sample
	C.kr. 48	Blood sample
	C.kr. 39	Drain swab
*Candida parapsilosis (C.p.)*	22019 ATCC	Reference
	C.p. 45	Blood sample
	C. p. 47	Blood sample
	C.p. 54	Blood sample
	C.p. 56	Blood sample
	C.p. 98	Blood sample
	C.p. 43	Blood sample from central venous catheter
	C.p. 46	Central venous catheter swab
	C.p. 55	Central venous catheter swab
	C.p. 71	Drain swab
	C.p. 166	Oral cavity swab
	C.p. 120	Ulcer swab
*Candida tropicalis (C.t.)*	C.t. 53	Blood sample
	C.t. 6	Central venous catheter swab
	C.t. 1	Cerberospinal fluid
	C.t. 68	Drain swab
	C.t. 72	Drain swab
	C.t. 150	Sputum sample
	C.t. 152	Tongue swab
	C.t. 168	Urine sample
	C.t. 80	Wound swab

### Antifungal susceptibility testing and evaluation of interaction with compound 4

Antifungal susceptibility to compound **4** was determined for each strain using the Method M27-A3, CISI, 2008. The final inoculum, ranging from 0.5 × 10^2^ to 2.5 × 10^3^ cfu/mL was prepared in synthetic RPMI 1640 medium (Gibco®, UK). The compound test wells (PTW) were prepared with stock solution of compound **4** (1600 μg/mL) dissolved in water 66% DMSO. Subsequently, serial two-fold dilutions were performed with RPMI 1640 medium. Then, the yeast cells suspension and compound **4** (final dilution 1:100) were dispensed at final volume of 250 μL/well into 96-well microplates. The compound **4** was tested at concentrations that ranged from 0.00195 to 32 μg/mL. As control, DMSO was tested at concentration of 0.66% (v/v) in antifungal susceptibility testing. At this concentration, DMSO was not able to inhibit the fungal growth. Growth control wells (GCW; containing medium, inoculum, the same amount of DMSO used in PTW, but compound-free) and sterility control wells (SCW; sample, medium, and sterile water replacing inoculum) were included for each strain tested. Microplates, after 18 and 48-h incubation at 35°C, were read with the Spark Control M10 (Tecan Group Ltd., Austria). Percentage of cell inhibition was read spectrophotometrically (optical density OD_405_) and the endpoint was calculated as a 100% reduction in OD_405_ as compared to the growth in control wells. Growth reduction for each compound concentration was calculated as follows: % of inhibition = 100 − (OD_405_ PTW − OD_405_ SCW)/(OD_405_ GCW − OD_405_ SCW). Additionally, the range of Amphotericin B (Sigma-Aldrich, USA) concentrations tested was 0.0195–2.5 μg/mL against *C. albicans* 90028. These concentrations were diluted in RPMI and were subsequently used in the assay as the reference concentrations. Minimal inhibitory concentrations (MICs) of compound were read visually after 18 and 48 h after shaking incubation at 35°C. We used two endpoint criteria for MIC determination: (i) total inhibition (MIC_TI_), the lowest concentration of compound that yielded no visible growth (a clear well); and (ii) partial inhibition (MIC_PI_), the lowest concentration that produced a prominent decrease in turbidity compared to that of the drug–free control (visual assessment; Majoros et al., 2005). For determination of the minimal fungicidal concentration (MFC), we used the method described by Majoros et al. (2005). The MFC was defined as the lowest compound concentration that killed ≥99.9% of viable cells, compared with the starting inoculum (≥3-log reduction). Tests were performed in triplicate with randomly selected strains at the range of concentrations of compound **4** from 2 to 32 μg/mL. Briefly, 50-μL aliquots from the selected wells of plates were removed after 48 h of incubation at 35°C. Then after 1/10,000 dilutions, 100 μL aliquots were placed onto agent-free Sabouraud dextrose agar plates and incubated at 35°C for 48 h (Rueda et al., 2014). Paradoxical growth (PG), the reduced fungicidal effect at higher concentrations, was also evaluated in MFC assay. In this assay, Paradoxical growth was defined as killing activity (99.9% reduction of the starting inoculum) observed at = 2 drug dilutions above MIC, but consistent lack of killing by higher concentrations (Shields et al., 2011).

### Sorbitol assay—effect of compound 4 on the cell wall of *C. albicans*

The MIC of compound **4** was determined in the presence of an osmotic protector using the Method M27-A3, CISI, 2008. SDB medium was added to each well of the microplate and serial dilutions of compound **4** (2.08–1066 μg/mL) were carried. Finally, 100 μL of inoculum (0.5 × 10^2^–2.5 × 10^3^ cfu/mL) prepared in SDB medium supplemented with sorbitol at final concentration of 0.8 M (Sigma-Aldrich) was transferred to wells as described Pereira et al. (2016). Plates were incubated at 35°C and read after 2 and 7 days (Lima et al., 2013; Freires et al., 2014; Pereira et al., 2016).

#### Cytotoxicity assays *in vitro*

Vero cells (ATCC CCL-81, LGC Poland) were cultured in EMEM medium (10% FCS, 1 mM pyruvic acid, 100 U/mL penicillin and 0,1 mg/mL streptomycin, LGC Poland) at 37°C in a humidified atmosphere containing 5% CO_2_. Vero cells were seeded into a 96-well plate (2 × 10^4^ cells per well). After an overnight incubation at 37°C in 5% CO_2_, compound **4** was added to cells, respectively. A dilution series of compound **4** (256–0.125 μg/mL final concentrations) were then supplied to wells so that each well contained 200 μl of medium in total. The plate was then incubated at 37°C in a humidified atmosphere 5% CO_2_ for 18 h, and then the toxic effects of the extracts were assayed using the XTT (sodium 3′-[1-(phenyl amino-carbonyl)-3,4-tetrazolium]-bis-[4-methoxy-6-nitrobenzene sulfonic acid hydrate) cytotoxicity assay according to the manufacturer (Roche Diagnostics GmbH, Germany). Fifty micro-liters of XTT reagent was added to each well and incubated for 18 h. For comparison, DMSO was used at the proper concentration (see above). The concentration at which the cell viability had dropped by 50% was recorded as the cytotoxic concentration (CC_50_; Cavalcanti et al., 2012). The optical densities of wells were measured at 450 nm (690 nm reference wavelength) using BIOTEK ELX800 multi well reader.

### *In vivo* cytotoxicity testing of compound 4

*Galleria mellonella* (Lepidoptera: Pyralidae) caterpillars in the final instar larval stage were used in the study (Insua et al., 2013; Li et al., 2013). A Hamilton syringe with a 30-gage needle was used to deliver compound **4** at concentrations ranging from 32 to 0.25 μg/mL in volume of 10 μL into the haemocoel of each larva via the last pro-leg. Before injection, the area was cleaned using an alcohol swab. Ten randomly chosen caterpillars (231 ± 40 mg) were injected at each concentration of compound **4**. Two controls were employed in all assays: the first one consisted of larvae which were untouched, the second control contained larvae whose pro-leg was inoculated with DMSO to ensure that death was not due to solvent. Larvae were placed into Petri dishes and incubated in dark at 37°C in a stationary incubator (Mowlds and Kavanagh, 2008). The number of dead larvae was scored daily for up to 72 h after injection. Larval death was assessed by the lack of movement of larvae in response to physical stimuli with melanisation of the cuticle. Results represent the mean percentage survival of larvae following the combination of the data from all experiments.

### Confocal microscopy

The assessment of compound **4** influence on *C. albicans* morphogenesis during 18-h growth on Caco-2 monolayer was performed by using Fluoview FV10i Microscope (Olympus, USA). Briefly, Caco-2 cells grown on 12 mm glass slides (Thermo SCIENTIFIC, Germany) in 24-well plates were inoculated after 21-day post-seeding with 1–1.2 × 10^6^ log phase yeast cells/mL of the *C. albicans* 90028 strain. After 18-h incubation at 37°C in EMEM medium (10% FCS, 1 mM pyruvic acid, without antibiotics, or antifungal agents), *C. albicans* sessile cells were treated with compound **4** at 16 μg/mL for 18 h. Amphotericin B at concentration of 1 μg/mL was used as a control. Then, slides were gently removed and washed three times with PBS to remove non-adherent *C. albicans* cells, and samples were stained with Propidium Iodide (PI, Roche Diagnostics GmbH), according to manufacturer's instruction. Untreated control cells were stained with Acridine Orange to show Caco-2 monolayer. Experiments were performed in duplicate and repeated at least three times.

### Antifungal activity of compound 4 against *Candida albicans* biofilms

Biofilms were formed 96-well plates as previously described (Pierce et al., 2010). After biofilm formation, the medium was discarded and non-adherent cells were removed by washing the biofilms three times with PBS. The highest working concentration of compound **4** (256 μg/mL) prepared from the stock solution of 1600 μg/mL in RPMI 1640 medium was added to wells in column one of plates containing fungal biofilms. Then, the series of doubling dilutions of compound **4** were created. Untreated biofilms containing RPMI 1640 and wells containing medium without biofilm were included as positive and negative controls, respectively. The plates were incubated at 37°C for 24 h without shaking. The antifungal effect was monitored by using the metabolic 2,3-bis(2-metoxy-4-nitro-5-sulfophenyl)-2H-tetrazolium-5-carboxanilide (XTT; Roche Diagnostics GmbH) reduction assay, with optical density being read at 490 nm (Bachmann et al., 2002; Pierce et al., 2010). Eight replicate biofilms were included for each experiment, and repeated on two separate occasions.

### Statistical analysis

Each experiment was performed at least three times, with experimental values expressed as means ± SD. The statistical differences between the control and test values were determined by means of the Wilcoxon signed-rank matched-pair test. Two-tailed *P* ≤ 0.05 were considered to be statistically significant.

## Results

### Antifungal susceptibility profile of *Candida* spp. to compound 4

**Figure 2:**
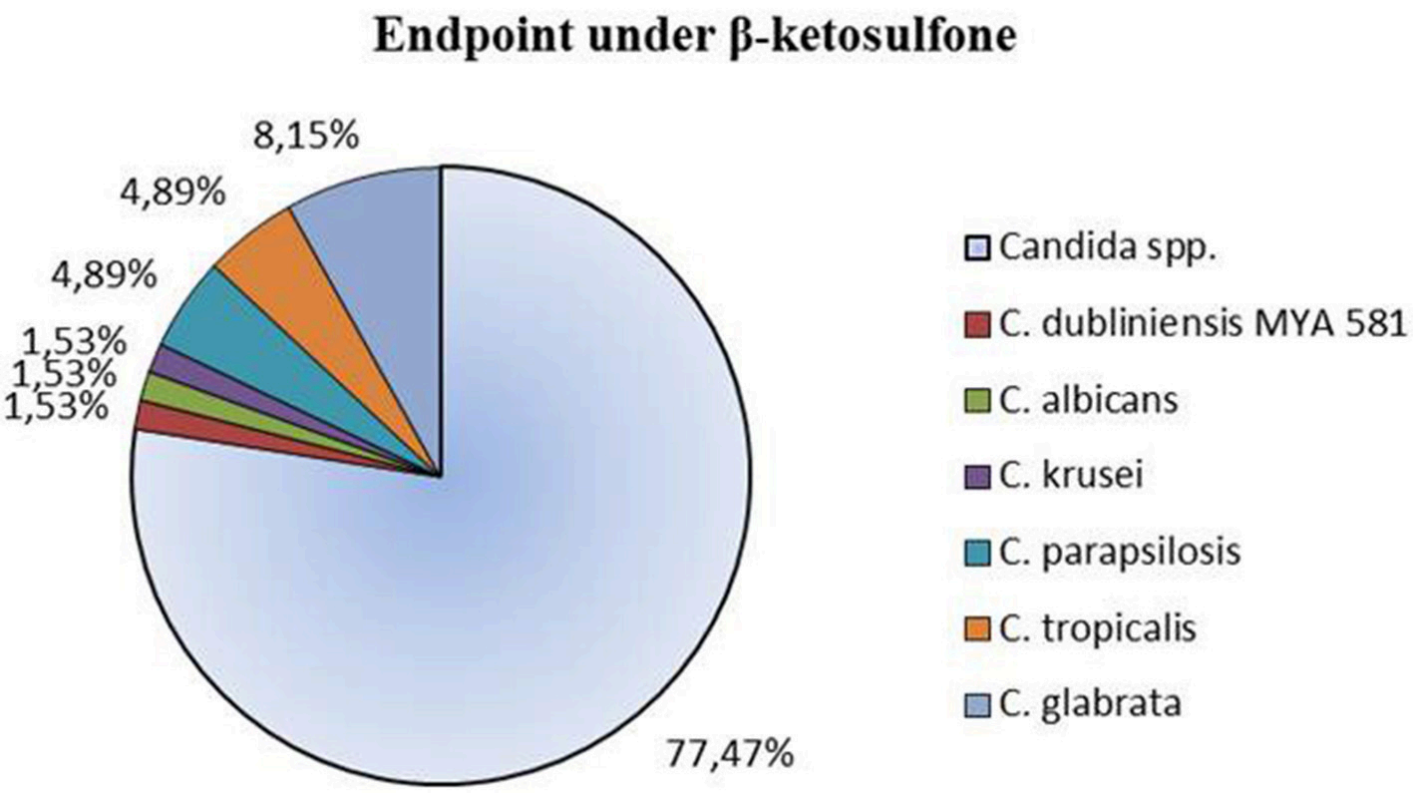
**Clear endpoint of the clinical strains of ***Candida*** spp. treated with compound 4 (β-ketosulfone) for 48 h in the microdilution assay**. Results are expressed as percentages of total number of strains recorded for the antifungal susceptibility profile study. Assays were performed on 2 independent occasions with 3 biological repeats (*n* = 3).

*In vitro* susceptibility testing to compound **4** was performed for 63 isolates. Percentage of growth inhibition was determined, respectively, for 38 isolates at concentrations ranging from 16 to 0.5 μg/mL (Table 2) and for 25 strains at 32–0.00195 μg/mL (Table 3). The proportion of strains that showed clear endpoint was 22% (14 out of 63 strains) in the two experiments (Figure 2). Compound **4** displayed a promising anti-*Candida* activity against *C. glabrata* and *C. tropicalis* (100% of growth inhibition ranged from 0.25 to 16 μg/mL). Among 17 *C. glabrata* isolates that are evaluated in the present study, 29% (5 out of 17) of strains showed a clear endpoint. For *C. tropicalis*, 33% of the isolates (3 out of 9 strains) were totally inhibited (clear endpoint). Although, endpoint was visible mostly for the latter strains at the range: 16–0.5 μg/mL compared with the remaining strains tested, it was evident that this effect appeared for *C. krusei* after the extension of the concentration tested to 0.00195 μg/mL (0.25 μg/mL = 100%). Unfortunately, compound **4** displayed anti-*Candida* activity (without clear endpoint) against most of the clinical strains of *C. albicans* (76%). We observed the presence of a visually assessed MIC in compound **4** with susceptibility testing plates for 35% of strains (22 out of all 63 strains tested). The proportion of strains that showed MIC was as follows: 14% (3 out of 22 strains) at concentrations: 0. 00195 and 0.0156 μg/mL; 23% (5 out of 22) at 0.0039 μg/mL; 27% (6 out of 22 strains) at 0.0078 μg/mL; as well as 4% (1 out of 22 strains) at concentrations: 0.0313–0.0625, 0.5–1, and 32 μg/mL. We found that the MIC values assessed visually corresponded to a higher percentage of growth inhibition in wells (measurement at OD_405_) compared to the remaining wells in a microdilution assay (Table 3). We observed that under these conditions the MIC value for one representative-reference strain of *C. albicans* 90028 was extremely high at 32 μg/mL. The MIC value of compound **4** against 90028 was significantly higher than that of AmB (MICx128 of AmB). MIC range among most of the clinical strains of *C. albicans* was 0.00195–0.0078 μg/mL. Although, we observed MIC only in 22 strains among the whole group tested, this experiment was not affected by the compound precipitation on well bottoms as compared to SCW.

**Table 2:** **Antifungal activity (cells inhibition %; Means ± SD) of compound 4 against ***Candida*** clinical isolates after 48 h**.

**Organism**	**Origin**	**Strain designation**	μ**g/mL**						
			**16**	**8**	**4**	**2**	**1**	**0.5**	0.625^*^
*Candida albicans*(Ca)	Blood sample	Ca no. 82	98.28 ± 0.17	98.44 ± 0.42	99.03 ± 0.47	99.04 ± 0.58	99.02 ± 0.29	99.10 ± 0.51	100
		Ca no. 129	98.73 ± 0.77	98.65 ± 0.76	98.68 ± 0.53	98.88 ± 0.19	98.91 ± 0.06	99.03 ± 0.04	100
		Ca no. 171	98.78 ± 0.14	98.74 ± 0.01	98.95 ± 0.70	99.13 ± 0.10	99.01 ± 0.20	98.92 ± 0.85	100
	Drain swab	Ca no. 87	99.13 ± 0.13	98.37 ± 1.86	98.78 ± 0.35	99.31 ± 0.58	98.96 ± 0.21	98.95 ± 0.98	100
	Nose swab	Ca no. 96	98.69 ± 0.83	99.06 ± 0.11	99.02 ± 0.08	99.01 ± 0.16	99.00 ± 0.09	99.11 ± 0.26	100
		Ca no. 97	98.99 ± 0.06	98.94 ± 0.13	99.03 ± 0.06	98.83 ± 0.17	98.98 ± 0.05	98.94 ± 0.24	100
	Sputum sample	Ca no. 128	98.83 ± 0.04	98.90 ± 0.29	98.64 ± 0.65	98.98 ± 0.36	98.91 ± 0.46	98.85 ± 0.51	100
	Vaginal swab	Ca no. 16	98.97 ± 0.19	98.82 ± 0.23	98.78 ± 0.32	98.98 ± 0.14	99.14 ± 0.21	99.49 ± 0.62	100
		Ca no. 28	98.20 ± 0.17	98.66 ± 1.03	98.58 ± 1.07	99.25 ± 0.31	99.05 ± 0.49	99.08 ± 0.26	100
	Wound swab	Ca no. 5	98.82 ± 0.11	98.89 ± 0.21	98.88 ± 0.14	98.88 ± 0.14	98.91 ± 0.13	98.86 ± 0.27	100
		Ca no. 89	98.62 ± 0.17	98.77 ± 0.24	99.01 ± 0.22	99.04 ± 0.30	98.78 ± 0.31	99.10 ± 0.20	100
*Candida dubliniensis*(Cd)	Reference	Cd ATCC MYA-581	99.88 ± 3.59	100 ± 0.91	99.55 ± 0.87	98.76 ± 0.79	99.95 ± 0.47	100 ± 2.08	100
*Candida glabrata* (Cg)	Anus swab	Cg no. 118	100 ± 3.92	100 ± 0.74	100 ± 0.61	100 ± 2.57	99.18 ± 1.45	99.76 ± 1.27	100
	Drain swab	Cg no. 4	99.05 ± 0.15	98.98 ± 0.26	98.64 ± 0.47	98.78 ± 0.21	98.73 ± 0.18	99.12 ± 0.18	100
		Cg no. 40	100 ± 1.36	100 ± 2.94	99.06 ± 1.92	98.88 ± 0.98	97.67 ± 1.38	99.61 ± 0.54	100
		Cg no. 70	99.17 ± 0.25	98.63 ± 0.34	99.04 ± 0.27	99.16 ± 0.01	98.77 ± 0.31	98.87 ± 0.26	100
		Cg no. 88	98.47 ± 1.86	98.64 ± 0.36	98.44 ± 0.51	98.51 ± 0.33	100 ± 4.78	98.59 ± 1.59	100
	Nose swab	Cg no. 67	99.11 ± 0.32	98.34 ± 0.35	98.87 ± 0.54	99.11 ± 0.35	98.98 ± 0.48	99.16 ± 0.15	100
		Cg no. 69	98.99 ± 0.18	98.97 ± 0.46	98.91 ± 0.02	99.10 ± 0.26	98.99 ± 0.33	99.00 ± 0.24	100
	Oral cavity swab	Cg no. 117	99.21 ± 1.20	97.92 ± 5.45	95.11 ± 4.48	98.58 ± 0.92	98.44 ± 0.06	98.53 ± 0.54	100
	Stoma swab	Cg no. 41	96.95 ± 1.44	98.80 ± 1.01	99.43 ± 0.47	100 ± 4.17	100 ± 1.01	100 ± 0.26	100
	Wound swab	Cg no. 73	98.83 ± 0.21	99.05 ± 0.42	99.00 ± 0.36	98.85 ± 0.06	99.17 ± 0.06	98.94 ± 0.26	100
		Cg no. 75	99.05 ± 0.76	98.70 ± 1.18	97.40 ± 5.36	98.56 ± 0.48	98.22 ± 0.92	98.81 ± 0.58	100
*Candida kefyr* (Ck)	Vaginal swab	Ck no. 27	99.24 ± 0.32	98.61 ± 0.85	99.30 ± 0.24	99.29 ± 0.93	98.62 ± 0.91	98.82 ± 0.70	100
*Candida krusei* (Ckr)	Bile sample	Ckr no. 119	98.66 ± 0.23	98.47 ± 0.68	99.23 ± 0.26	99.19 ± 0.04	98.98 ± 0.29	99.09 ± 0.29	100
*Candida parapsilosis* (Cp)	Blood sample	Cp no. 56	99.79 ± 1.06	99.39 ± 5.11	97.46 ± 1.16	98.97 ± 0.51	98.70 ± 0.46	100 ± 0.65	100
		Cp no. 98	99.00 ± 0.17	99.10 ± 0.24	98.94 ± 0.26	99.28 ± 0.11	98.82 ± 1.03	99.00 ± 0.29	100
	Central venous catheter swab	Cp no. 55	98.85 ± 0.17	98.92 ± 0.22	98.97 ± 0.34	99.06 ± 0.16	98.88 ± 0.22	99.08 ± 0.20	100
	Drain swab	Cp no. 71	98.77 ± 1.07	98.98 ± 0.09	98.94 ± 0.20	98.98 ± 0.17	98.91 ± 0.17	98.83 ± 0.40	100
	Oral cavity swab	Cp no. 166	98.71 ± 0.23	99.11 ± 0.24	98.90 ± 0.17	98.84 ± 0.09	98.94 ± 0.09	98.96 ± 0.13	100
	Ulcer swab	Cp no. 120	98.85 ± 0.22	99.05 ± 0.45	99.07 ± 0.43	99.05 ± 0.21	99.05 ± 0.46	99.03 ± 0.32	100
*Candida tropicalis* (Ct)	Blood sample	Ct no. 53	83.13 ± 0.00	100 ± 2.12	99.32 ± 1.77	99.78 ± 0.87	99.19 ± 0.23	100 ± 0.66	100
	Central venous catheter swab	Ct no. 6	100 ± 0.00	100 ± 0.00	100 ± 0.24	99.23 ± 0.88	97.76 ± 9.04	98.27 ± 9.11	100
	Drain swab	Ct no. 68	98.98 ± 0.31	98.98 ± 0.15	99.03 ± 0.21	99.09 ± 0.26	99.01 ± 0.12	99.00 ± 0.16	100
	Drain swab	Ct no. 72	99.89 ± 0.03	99.70 ± 0.64	93.67 ± 12.52	96.61 ± 11.35	98.97 ± 0.55	98.91 ± 0.29	100
	Sputum sample	Ct no. 150	99.00 ± 0.04	99.07 ± 0.49	98.94 ± 0.54	98.93 ± 0.19	99.03 ± 0.07	98.94 ± 0.18	100
	Urine sample	Ct no. 168	98.94 ± 0.01	98.98 ± 0.13	98.96 ± 0.30	99.03 ± 0.08	99.04 ± 0.07	98.63 ± 1.42	100
	Wound swab	Ct no. 80	99.09 ± 0.23	98.99 ± 0.24	99.14 ± 0.41	98.84 ± 0.16	98.87 ± 0.13	99.01 ± 0.29	100

* *stands for MIC of Amphotericin B (AmB) determined using the broth microdilution Method M27-A3, CISI, 2008, for AmB at the concentration of 0.625 μg/mL against all strains tested clear end point (100% growth reduction) was determined spectrophotometrically (optical density OD_405_)*.

**Table 3:** **Antifungal activity (cells inhibition%; Means ± SD) of compound 4 against ***Candida*** clinical isolates after 48 h**.

**Organism**	**Origin**	**Strain designation**	**μg/mL**														
			**32**	**16**	**8**	**4**	**2**	**1**	**0.5**	**0.25**	**0.125**	**0.0625**	**0.0313**	**0.0156**	**0.0078**	**0.0039**	**0.00195**
*C. albicans(C.a.)*	Reference	C. a. 90028ATCC	99.78 ± 0.56	99.83 ± 0.13	99.39 ± 0.32	99.26 ± 0.19	98.78 ± 0.61	99.10 ± 0.60	98.82 ± 0.36	99.13 ± 2.9	99.19 ± 4.26	98.96 ± 0.55	98.66 ± 0.35	98.95 ± 0.23	98.29 ± 0.36	98.76 ± 0.69	98.43 ± 6.01
		C.a. 14053 ATCC	98.99 ± 0.01	98.97 ± 1.00	99.98 ± 0.00	98.98 ± 0.02	98.96 ± 0.03	99.98 ± 0.07	99.22 ± 0.33	99.29 ± 0.47	99.61 ± 0.43	99.76 ± 0.35	99.67 ± 0.55	99.25 ± 0.29	**99.68** ± **0.42**	99.13 ± 0.07	99.34 ± 0.45
		C.a. 28836 ATCC	99.95 ± 0.02	98.90 ± 0.00	98.89 ± 0.00	98.76 ± 0.0.05	98.81 ± 0.05	99.20 ± 0.55	99.26 ± 0.50	99.98 ± 0.01	98.92 ± 0.14	99.55 ± 0.58	99.30 ± 0.22	98.92 ± 0.25	**99.57** ± **0.55**	99.44 ± 0.40	99.09 ± 0.30
	Stool sample	C. a. 131	99.79 ± 0.23	99.15 ± 0.51	99.27 ± 0.38	99.25 ± 0.38	99.13 ± 0.16	98.92 ± 0.03	99.05 ± 0.01	99.40 ± 0.52	99.58 ± 0.55	77.66 ± 6.45	99.60 ± 0.48	99.35 ± 0.43	**99.65** ± **0.45**	99.00 ± 0.01	98.99 ± 0.03
	Vaginal swab	C.a. 15	99.32 ± 0.46	92.82 ± 6.14	99.11 ± 0.18	99.08 ± 0.22	98.95 ± 0.02	99.01 ± 0.02	99.04 ± 0.08	99.63 ± 0.58	99.34 ± 0.45	99.51 ± 0.36	99.64 ± 0.47	99.33 ± 0.45	99.23 ± 0.10	**99.83** ± **0.20**	98.98 ± 0.06
		C.a. 18	99.98 ± 0.01	99.34 ± 0.27	99.40 ± 0.23	98.99 ± 0.27	99.38 ± 0.45	99.09 ± 0.25	99.55 ± 0.28	99.08 ± 0.93	99.56 ± 0.15	99.59 ± 0.20	82.19 ± 5.77	99.84 ± 0.05	99.64 ± 0.23	99.83 ± 0.03	**99.68** ± **0.03**
	Wound swab	C.a. 86	99.94 ± 0.00	99.11 ± 0.16	99.44 ± 0.19	79.08 ± 19.89	98.73 ± 0.13	99.05 ± 0.05	88.34 ± 10.65	99.18 ± 1.53	99.15 ± 0.11	99.44 ± 0.01	99.29 ± 0.08	99.91 ± 0.10	**99.95** ± **0.00**	99.60 ± 0.49	99.40 ± 0.79
*C. glabrata(C.g)*	Aspirate sample	C.g. 144	99.71 ± 0.00	99.71 ± 0.00	99.71 ± 0.00	99.71 ± 0.00	99.71 ± 0.00	99.71 ± 0.00	99.71 ± 0.00	99.54 ± 0.31	99.95 ± 0.01	99.94 ± 0.03	99.55 ± 0.30	**99.97** ± **0.00**	99.58 ± 0.27	99.78 ± 0.26	99.97 ± 0.47
	Blood sample	C.g. 44	99.87 ± 0.01	99.31 ± 0.48	99.33 ± 0.35	99.22 ± 0.20	99.20 ± 0.15	98.14 ± 0.93	98.39 ± 0.50	100 ± 0.44	99.78 ± 0.23	97.51 ± 0.89	97.66 ± 1.37	99.63 ± 0.27	**99.45** ± **0.15**	98.20 ± 0.23	98.71 ± 0.59
	Drain swab	C.g. 85	97.72 ± 0.89	98.24 ± 1.22	98.79 ± 0.42	98.59 ± 0.38	98.63 ± 0.90	97.48 ± 0.65	96.89 ± 2.17	98.60 ± 1.75	99.46 ± 0.49	99.60 ± 0.10	98.44 ± 0.67	98.85 ± 0.32	99.59 ± 0.31	99.270.56	99.50 ± 0.36
		C.g. 92	97.54 ± 0.92	99.28 ± 0.65	98.79 ± 0.09	98.91 ± 0.09	98.88 ± 0.37	98.86 ± 0.06	99.30 ± 0.51	99.54 ± 0.74	99.57 ± 0.04	99.02 ± 0.17	98.53 ± 0.88	99.16 ± 0.05	99.92 ± 0.02	99.01 ± 0.11	**99.91** ± **0.01**
		C.g. 99	99.75 ± 0.18	99.09 ± 0.02	91.25 ± 7.80	99.42 ± 0.50	98.32 ± 0.98	99.15 ± 0.01	99.38 ± 0.19	99.23 ± 0.21	99.91 ± 0.04	97.88 ± 2.66	88.00 ± 4.45	99.05 ± 0.22	97.73 ± 1.35	**99.51** ± **0.16**	98.48 ± 0.28
	Wound swab	C.g. 74	98.95 ± 0.23	99.36 ± 0.54	99.13 ± 0.11	98.64 ± 0.67	98.97 ± 0.23	99.30 ± 0.10	99.27 ± 0.22	99.01 ± 0.02	98.14 ± 1.46	98.98 ± 0.20	**99.33** ± **0.21**	99.13 ± 0.15	98.75 ± 0.47	98.91 ± 0.04	98.91 ± 0.03
*C. krusei(C.kr)*	Reference	C.kr. 6258 ATCC	99.95 ± 0.00	99.95 ± 0.01	99.97 ± 0.00	99.93 ± 0.03	99.91 ± 0.07	99.98 ± 0.00	99.85 ± 0.16	99.96 ± 0.01	99.70 ± 0.36	99.89 ± 0.11	99.92 ± 0.05	99.90 ± 0.10	**99.96** ± **0.06**	99.92 ± 0.02	99.97 ± 0.00
	Aspirate sample	C.kr. 122	99.88 ± 0.05	99.35 ± 0.63	99.19 ± 0.08	95.73 ± 2.87	99.10 ± 0.19	99.49 ± 0.12	99.26 ± 0.00	100 ± 0.37	99.65 ± 0.57	99.68 ± 0.47	99.79 ± 0.30	99.28 ± 1.05	98.66 ± 0.33	**99.93** ± **0.01**	98.41 ± 2.10
	Blood sample	C.kr. 48	99.81 ± 0.07	99.48 ± 0.36	98.53 ± 0.47	99.14 ± 0.05	98.76 ± 0.36	98.80 ± 0.27	98.80 ± 0.44	98.42 ± 0.35	99.32 ± 0.20	98.33 ± 0.27	99.35 ± 0.10	99.26 ± 0.27	99.02 ± 0.52	98.74 ± 0.78	99.12 ± 0.17
	Drain swab	C.kr. 39	99.46 ± 0.44	97.93 ± 1.18	99.49 ± 0.31	99.39 ± 0.13	99.08 ± 0.32	99.39 ± 0.05	99.53 ± 0.09	96.85 ± 2.10	99.60 ± 0.09	99.89 ± 0.11	99.84 ± 0.16	**99.91** ± **0.01**	99.39 ± 0.60	99.54 ± 0.27	89.40 ± 4.91
*C. parapsilosis(C.p.)*	Reference	C.p. 22019ATCC	99.41 ± 0.66	99.48 ± 0.43	99.38 ± 0.41	98.60 ± 0.77	98.23 ± 1.21	99.10 ± 0.00	98.35 ± 0.57	99.18 ± 0.52	99.16 ± 0.96	97.24 ± 3.48	96.41 ± 0.62	93.97 ± 2.67	99.93 ± 0.01	99.87 ± 0.02	**99.89** ± **0.02**
	Blood sample	C.p. 45	87.18 ± 5.13	99.51 ± 0.43	99.43 ± 0.43	99.10 ± 0.09	99.47 ± 0.48	99.32 ± 0.30	99.09 ± 0.10	100 ± 1.45	99.34 ± 0.89	**99.66** ± **0.11**	98.53 ± 1.67	94.25 ± 3.10	86.17 ± 5,79	99.07 ± 0.41	99.50 ± 0.68
	Blood sample	C.p. 47	97.92 ± 0.79	98.90 ± 0.07	98.25 ± 0.66	98.69 ± 0.25	99.19 ± 0.23	98.50 ± 0.47	98.13 ± 0.91	98.87 ± 0.55	99.15 ± 0.11	99.02 ± 0.25	99.34 ± 0.19	99.07 ± 0.68	98.79 ± 0.66	99.30 ± 0.19	99.13 ± 0.29
	Blood sample	C.p. 54	98.87 ± 0.01	98.78 ± 0.29	99.54 ± 0.42	98.98 ± 0.01	98.68 ± 0.03	**99.02** ± **0.14**	98.97 ± 0.09	99.10 ± 0.10	99.11 ± 0.07	99.11 ± 0.02	97.88 ± 1.53	99.05 ± 0.07	99.09 ± 0.14	99.00 ± 0.07	99.06 ± 0.01
	Blood sample from central venous catheter	C.p. 43	98.97 ± 0.13	99.02 ± 0.08	98.98 ± 0.09	88.84 ± 10.22	99.11 ± 0.13	99.01 ± 0.09	**99.45** ± **0.48**	97.48 ± 0.35	99.67 ± 0.43	99.41 ± 0.46	98.81 ± 0.50	97.62 ± 0.33	98.78 ± 0.41	99.24 ± 0.62	99.22 ± 0.05
	Central venous	C.p. 46	99.80 ± 0.03	98.00 ± 0.98	100 ± 0.09	99.98 ± 0.02	99.99 ± 0.02	100 ± 0.00	99.90 ± 0.09	99.78 ± 0.34	99.99 ± 0.03	99.84 ± 0.22	99.93 ± 0.06	99.98 ± 0.01	99.91 ± 0.07	**99.97** ± **0.03**	99.78 ± 0.27
*C. tropicalis(C.t.)*	Cerbero spinal fluid	C.t. 1	98.23 ± 0.37	98.03 ± 0.22	98.91 ± 0.10	98.83 ± 0.12	99.16 ± 0.19	99.13 ± 0.15	99.17 ± 0.10	98.98 ± 0.06	98.91 ± 0.10	99.89 ± 0.00	99.29 ± 0.03	99.14 ± 0.02	99.65 ± 0.09	**99.03** ± **0.08**	98.73 ± 0.20
	Tongue swab	C.t. 152	97.38 ± 2.45	97.06 ± 2.54	94.65 ± 5.34	60.71 ± 39.24	98.29 ± 0.67	99.98 ± 0.02	86.53 ± 11.62	100 ± 1.85	99.73 ± 0.04	98.79 ± 1.14	99.97 ± 0.01	**99.97** ± **0.02**	92.58 ± 3.78	99.06 ± 0.06	98.67 ± 0.03

*The percentage values given in the table are mean of three measurement of optical density at each concentration against all strains tested (mean ± SD for n = 3); The percentage values represents growth inhibition calculated as described in Materials and Methods, where cells treated with each concentration of compound **4** (2.5 μL per well) ranging from 0.00159–32 μg/mL were compared to growth control—GCW containing DMSO solvent (2.5 μL per well) at 0.66%; Bold stands for MIC assessed visually after 48 h of incubation with compound **4***.

We examined if compound **4** was fungicidal against clinical strains or if, contrariwise, it did not kill the cells and behaved as a fungistatic agent against these strains. For this purpose after growing the cells in compound **4** at concentrations from 32 to 2 μg/mL, we passed them on YEPD agar plates (separately from compound **4**) and then assessed their antifungal susceptibility to compound **4**. We found that these concentrations have the fungistatic effect on the following strains: Ca 90028, Ca 15, Cg 85, Ck 48, Cp 47, and Cp 43 (Table 4). As we showed, the effect of compound **4** activity against the latter strains was stable within this concentration range. Furthermore, compound **4** at 32 μg/mL exhibited fungicidal activity against nine strains tested (Table 4).

**Table 4:** **Fungicidal activity of β-ketosulfone**.

**Strain designation**	***Candida* colony forming unites (cfu x10^4^) after treatment with** β**-ketosulfone (**μ**g/Ml)**					**Control *Candida* colony forming unites (cfu × 10^9^)**	**Logarithm reduction of** ***Candida*** **unites after** **β-ketosulfone 48-h influence**				
	**32**	**16**	**8**	**4**	**2**	**Untreated starting inoculum**	**32**	**16**	**8**	**4**	**2**
C. a. 90028 ATCC	125	147	111	128	216	36	3-log	3-log	3-log	3-log	3-log
C.a. 14053 ATCC	59	129	0	124	158	0.9	2-log^*^	1-log^*^	9-log^*^	1-log^*^	1-log^*^
C.a. 28836 ATCC	0	16	63	71	123	2	10-log	3-log	3-log	1-log	2-log
C. a. 131	0	0	128	24	96	2	10-log	10-log	2-log	2-log	2-log
C.a. 15	198	85	125	97	69	2	1-log	2-log	2-log	2-log	2-log
C.a. 86	0	228	335	715	226	1	10-log	1-log	1-log	1-log	1-log
C.g. 144	3	0	13	3	0	3	3-log^*^	10-log^*^	4-log^*^	3-log^*^	10-log^*^
C.g. 44	0	18	33	36	58	7	10-log	4-log	4-log	3-log	4-log
C.g. 85	294	276	315	295	294	2	2-log	2-log	2-log	2-log	2-log
C.g. 92	0	0	0	0	0	1	10-log	10-log	10-log	10-log	10-log
C.k. 6258 ATCC	0	0	6	2	25	0.2	9-log	9-log	2-log	3-log	1-log
C.k. 122	0	0	83	66	226	1	10-log	10-log	2-log	2-log	2-log
C.k. 48	237	148	162	82	104	0.017	<1-log	<1-log	<1-log	<1-log	<1-log
C.k. 39	6	0	0	48	35	1	4-log	10-log	10-log	3-log	3-log
C.p. 47	150	535	881	439	492	9	2-log	2-log	2-log	2-log	1-log
C.p. 43	145	197	97	146	146	2	2-log	2-log	2-log	2-log	2-log
C.p. 46	117	33	41	78	13	4	3-log^*^	4-log^*^	4-log^*^	4-log^*^	4-log^*^
C.t. 152	42	33	39	0	0	0.4	4-log	2-log	2-log	9-log	9-log

*Logarithm Reduction of Candida spp. Colony Forming Unites under β-ketosulfone Influence*.

* *stands for paradoxical effect evaluated in MFC assay performed in n = 3, where logarithm reduction of the colony number of each strain was compared to the starting inoculum grown in RPMI medium with 0.66% DMSO (dissolved in water v/v)*.

### Compound 4 paradoxical effects among *Candida* spp.

**Figure 3:**
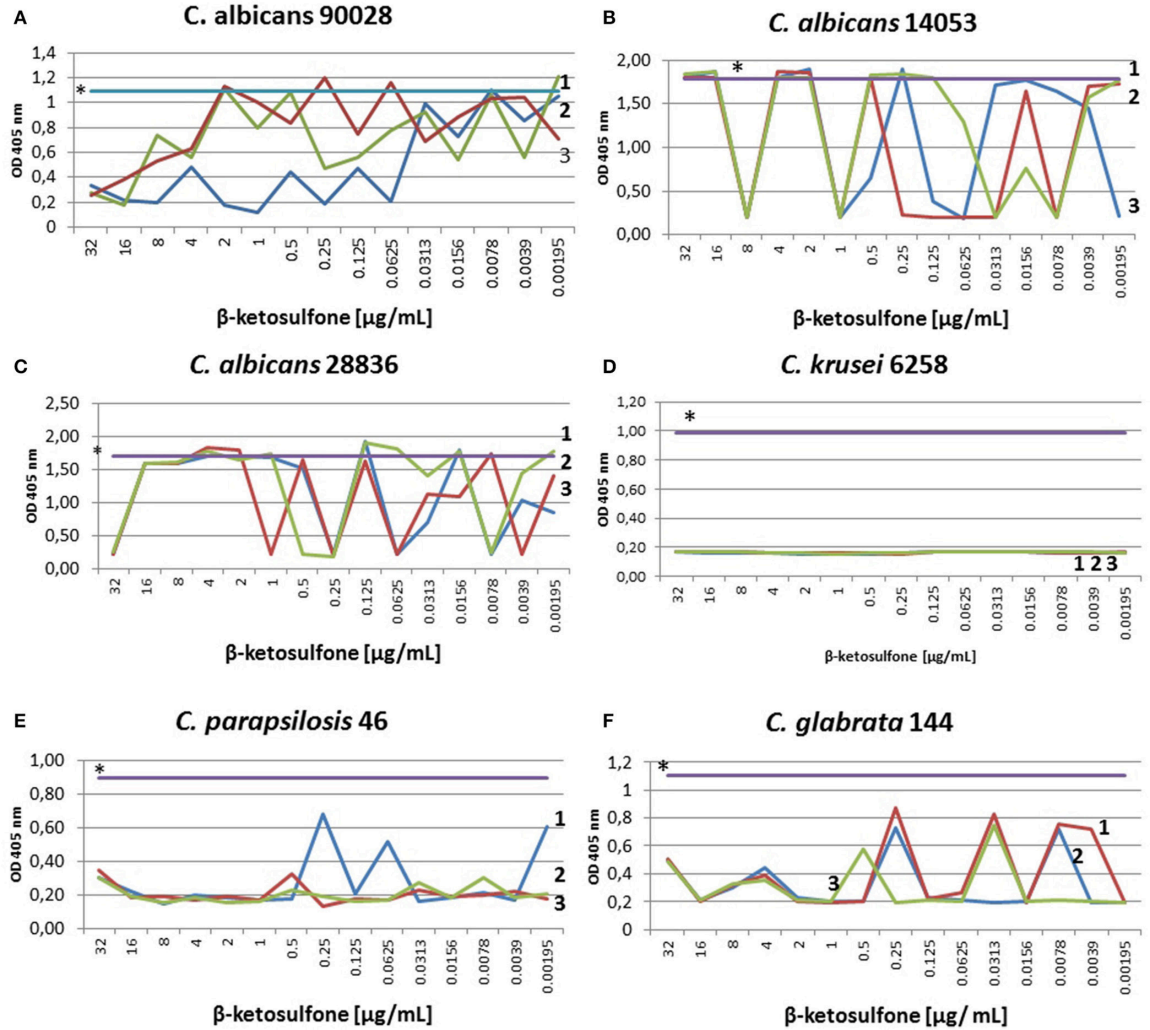
**Characterization of growth of ***Candida*** strains in the presence of compound 4**. Optical densities at 405 nm were obtained after treatment with different compound **4** concentrations. Control susceptibility test of compound **4** after 48 h against reference strains: *C. albicans* 90028 **(A)**, *C. albicans* 14053 **(B)**, *C. albicans* 28836 **(C)**, *C. krusei* 6258 **(D)**. Susceptibility test against the clinical strains: *C. parapsilosis* 46 **(E)** and *C. glabrata* 144 **(F)**. Testing antifungal susceptibility to compound **4** was performed as described in Materials and Methods after 48 h. Test was performed in 3 independent experiments. Results are presented as follows: ^*^growth control in RPMI with DMSO dissolved in water (0.66% v/v); 1, 2, 3 stand for three independent susceptibility tests. **(A)** The 48-h incubation with compound **4** yielded significantly reduced OD of 90028 at concentrations ranging from 16 to 32 μg/mL. No paradoxical growth in the presence of agent in *C. albicans* 90028 was noted. **(B)** Optical densities obtained after 48 h of incubation of *C. albicans* 14053 indicated paradoxical effect. The graph showed significantly decreased OD at 8 μg/mL, which correspond to 9-log reduction of cfu of *C. albicans* 14053 (Table 4). **(C)** The curves of ODs of *C. albicans* 28836 plotted against compound **4** concentrations showed higher starting OD and a steeper decrease in the slope of the curve at 32 μg/mL, which correspond to 10-log reduction (Table 4). **(D)** As determined by optical density, dramatically reduced viability of cells of *C. krusei* 6258 after exposure to compound **4** relative to the control grown in the presence of DMSO (0.66%) was seen. **(E)** *C. parapsilosis* 46 cells reduction after exposure to compound **4** correlated with end point at 8 and 1 μg/mL (Table 3), and 4-log reduction at concentrations ranging from 16 to 2 μg/mL (Table 4). Paradoxical growth in *C. krusei* 46 was noted. **(F)** Growth of cells of *C. glabrata* 144 incubated in the presence of 0.0156 μg/mL of compound **4** was significantly reduced, which corresponded to MIC (Table 3) and elevated reduction of cell growth (ranging from 4- to 10-log reduction at 8 and 16 μg/mL, respectively, Table 4).

We found a typical paradoxical effect, defined as growth in the presence of high concentrations of an antifungal agent and the absence of growth at intermediate concentrations for two clinical isolates tested: *C. glabrata* 144 and *C. parapsilosis* 46 (Table 4; Figure 3). We characterized this phenomenon in detail, firstly the percentage of growth inhibition being evaluated in the microdilution Method M27-A3, CISI, 2008 (Table 3). Although, the MIC value for strain 144 was 0.0156 μg/mL, we did not observe a clear endpoint at the concentration ranging from 32 to 0.00195 μg/mL. Contrariwise, strain 46 reached MIC at 0.0039 μg/mL and clear endpoints were observed at 1 and 8 μg/mL. These data indicated that paradoxical growth in the presence of compound **4** is not frequent among the *Candida* clinical isolates. We studied whether the growth characteristics were dependent on compound **4** concentrations compared to the control growth curves (Figure 3). The growth of control cells fluctuated in line with the DMSO concentration and strain. Compound **4** did not impair the final growth of yeast cells, since the cells were able to reach the final OD at the highest concentration (strain 144: 0.55 ± 0.004; strain 46: 0.35 ± 0.05) after 48 h (Figures 3E,F). In line with this, compound **4** at 32 μg/mL achieved only 3-log reduction of cells of both clinical isolates in the MFC assay (Table 4). The latter phenomenon was also noted for *C. albicans* 90028 (without paradoxical effect, Figure 3A). In the case of *C. albicans* 14053, 2-log cell reduction under compound **4** at the highest concentration tested was observed (Table 4). No difference between the final OD at 32 μg/mL (OD = 1.80 ± 0.02) and the growth control of strain 14053 (OD = 1.79 ± 0.009) was observed (Figure 3B). In line with the latter, complete killing of 14053 was not observed at the concentration of 32 μg/mL in MFC assay. The remaining references strains: *C. albicans* 28836 and *C. krusei* 6258 were reduced for 10- and 9-log, respectively (Table 4; Figures 3C,D).

### Effect of compound 4 on *C. albicans* cell wall

**Figure 4:**
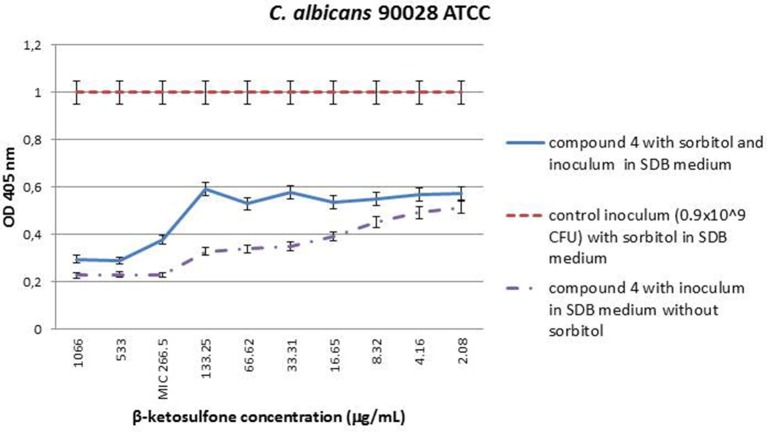
**MIC values (μg/mL) of β-ketosulfone (compound 4) in the presence of sorbitol (0.8 M) against ***C. albicans*** 90028**. The solutions of compound **4** were dissolved in water with 0.66% (v/v) addition of DMSO. The yeast cells suspension at final density of 0.9 × 10^9^ cfu/mL SDB medium with sorbitol at final concentration of 0.8 M and DMSO at 0.66% was used in the experiment. Additionally, to accurately determine MICs for 90028, compound **4** at the range of concentrations: 2.08–1066 in SDB medium without sorbitol was used.

The MIC value of compound **4** against strain 90028, in medium with sorbitol was seen at concentration of 266.5 μg/mL. The same effect was observed for compound **4** in medium without sorbitol. Cells protected with sorbitol grew in the presence of compound **4** at concentrations ranging from 2.08 to 133.25 μg/mL. The findings showed that compound **4** does not interfere with cell wall biosynthesis pathway because the susceptibility of yeast cells to it was unaltered in the presence or absence of an osmotic protector (Figure 4). The growth control ensured the viability of cells since they were able to grow in the presence of sorbitol and the absence of compound **4**.

### Compound 4 displays low toxicity to vero cells

**Figure 5:**
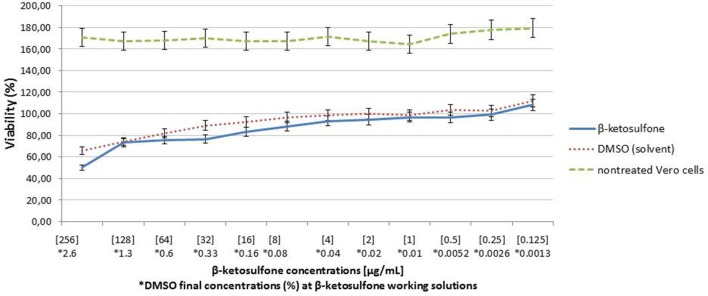
**Analysis of Vero cells viability after 18-h treatment with compound 4**. Cells viability was analyzed in parallel with DMSO (diluent).

Since Vero cells are the most common tool to assess influence of chemical and other compounds on epithelial cells (Ammerman et al., 2008), we employed them in our study to estimate the compound **4**′ cytotoxicity. Compound **4** at the highest concentration tested (256 μg/mL) exhibited almost 50% inhibition of Vero cells viability (CC_50_) after 18-h treatment (Figure 5). Cytotoxicity assays performed in 96-well plate revealed >CC_20_ of compound **4** at 64 and 32 μg/mL, respectively (Figure 5). At the concentration of 16 μg/mL, compound **4** caused a reduction of 16.49% in cells viability. Treatment considering DMSO (solvent) at 2.6% (Figure 5) caused approximately 12.26% reduction in the Vero cells viability.

#### Citotoxicity of compound 4 against *G. mellonella*

**Figure 6:**
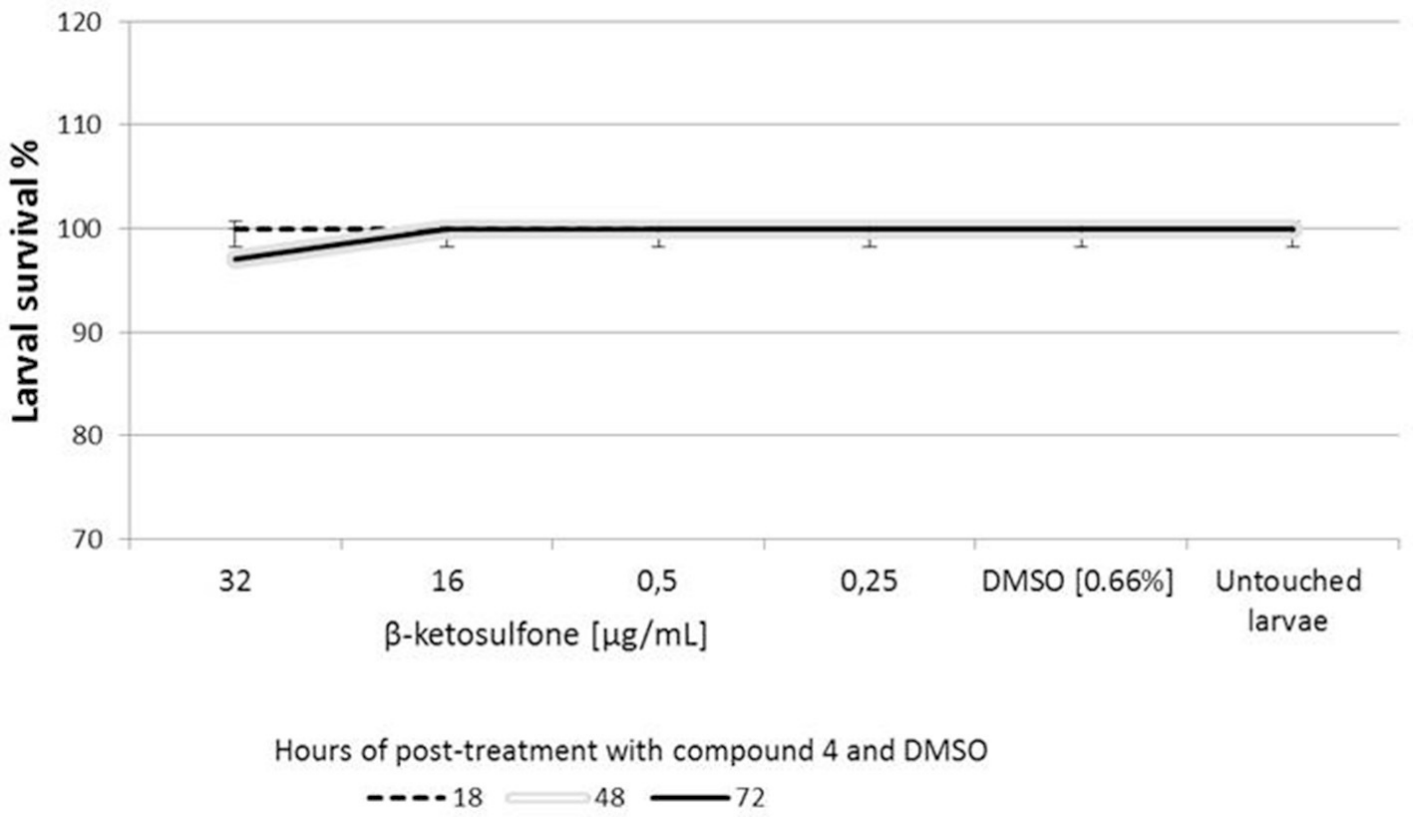
**Cytotoxicity of compound 4 against ***Galleria mellonella*****. Ten larvae were injected with compound **4** or DMSO (solvent used as a control) and incubated at 37°C for 72 h. The time of death of larvae was recorded after 18, 48, and 72 h of the post-treatment. Untouched larvae were used as a control of injection stress. Results are the mean of three separate experiments, ± standard deviation.

*In vivo* cytotoxicity test indicated that almost all larvae survived when inoculated with compound **4**. Low level of larval death occurred in the one set (per three tested for each concentration) of compound **4** at 32 μg/mL. The latter may have result from the unavoidable handling of larvae in the initial stages of the assays. The results are presented in Figure 6. We evaluated the toxicity of DMSO (solvent of compound **4**) using *G. mellonella*. We found that DMSO does not induce toxicity against larvae that contribute to the lack of toxicity evident following compound **4** challenges (Figure 6). The level of DMSO-injected and untouched insects remained constant (100% survival). Compound **4** injected larvae were not significantly different from those of DMSO-challenged and untouched insects at all-time points analyzed (Figure 6). We did not observe the dark color of larvae associated with the pigment melanin, an immune response by the insect to compound **4**.

### Antifungal susceptibility of *C. albicans* biofilm to compound 4

**Figure 7:**
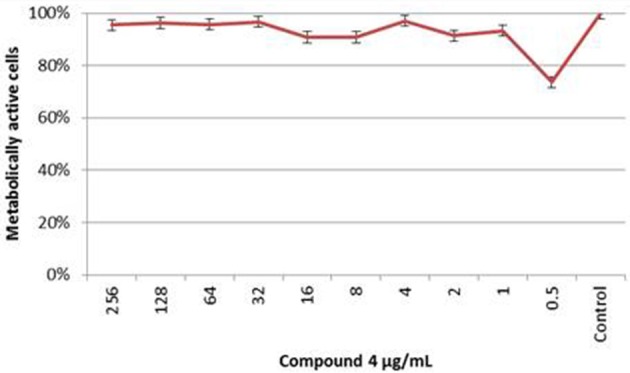
**The XTT Assay Results of the ***C. albicans*** 90028 Biofilm Susceptibility to Compound 4 At each concentration of compound 4, the metabolic activity of biofilm was compared with the control biofilm of 90028 (***p*** > 0.05)**.

The results obtained by XTT assay at 24 h are summarized in Figure 7. The effect of Compound **4** on formed *C. albicans* biofilms was minimal. Compound **4** at concentration of 0.5 μg/mL inhibited the metabolic activity of cells within biofilms but insignificant reduction was noted (<50%). A similar trend was observed for the range of concentrations 2–1 and 16–8 μg/mL. Furthermore, XTT assay denoted clear metabolic activity of sessile cells in the presence of compound **4** at concentrations above the MIC_PI_ (determined in broth microdilution method). Mean differences between the test and control assays (100% growth) were not statistically significant (*p* > 0.05).

### Influence of compound 4 on *C. albicans* morphogenesis during sessile growth on Caco-2 monolayer

**Figure 8:**
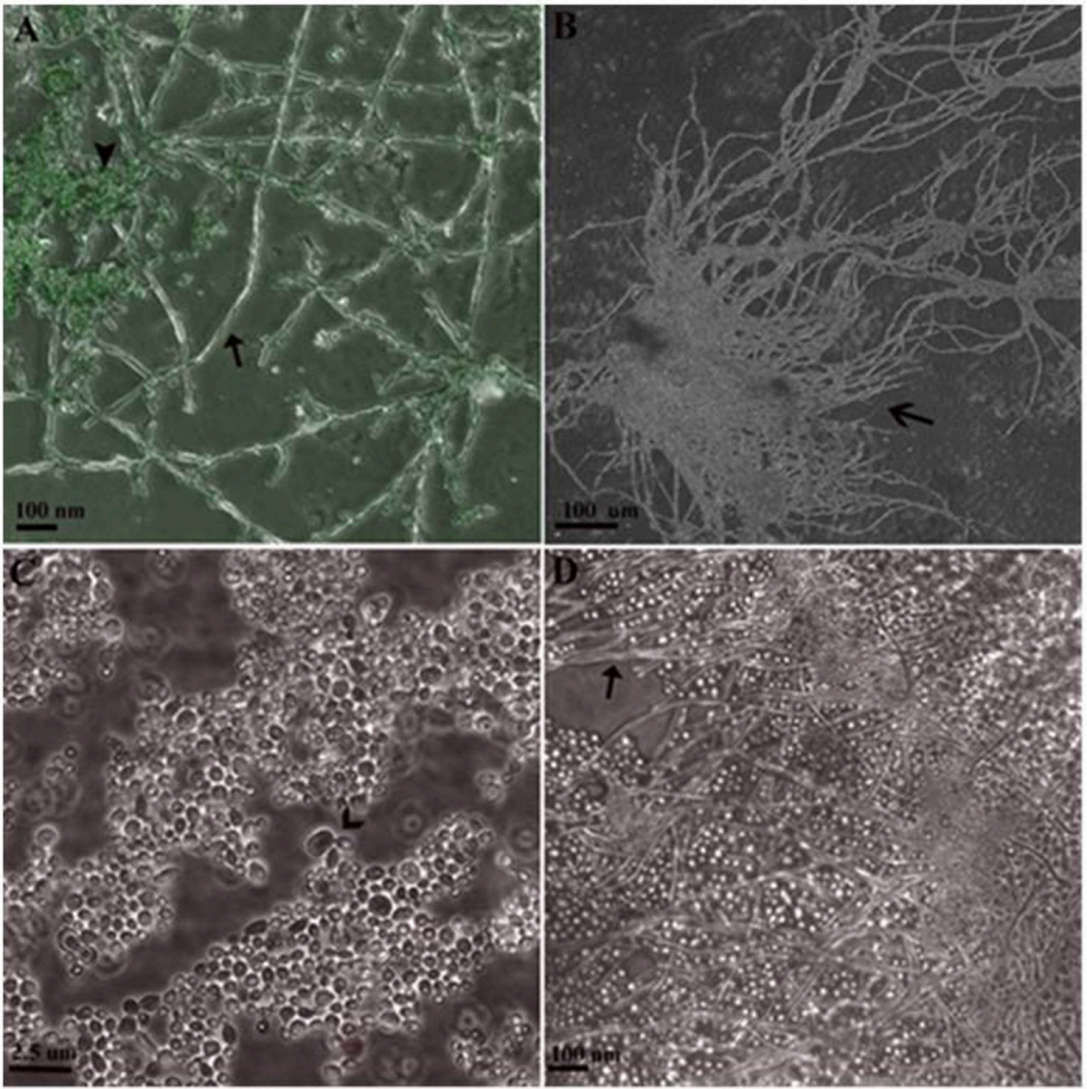
**Sessile Growth of ***C. albicans*** 90028 on slides with Caco-2 monolayer in 24-well plate**. **(A)** Hyphal cells not treated with antifungals (arrow) grown on cell line stained with acridine orange (arrowhead). **(B)** Conglomerate of hyphal forms (open arrow). **(C)** Under influence of Amphotericin B at 1 μg/mL for 18 h, budding blastoconidial cells were seen (open arrow) with lack of true hyphae. **(D)** After 18-h influence of compound **4** at 16 μg/mL, hyphal forms were observed (arrow).

The effect of compound **4** at MIC = 16 μg/mL on *C. albicans* morphogenesis during the interaction with Caco-2 cells was visually determined (Figure 8). Strain 90028 exhibited no defects in hyphal growth on Caco-2 monolayer. Thus, compound **4** at 16 μg/mL had minor or none effect on sessile *C. albicans* filament growth. As control, we observed a significant hypha inhibition under Amphotericin B at 1 μg/mL after 18-h treatment.

## Discussion

Here we synthesized and characterized compound **4**, the first, to our knowledge, β-ketosulfone exhibiting fungicidal activity. In our study, novel compound **4** was shown to possess effective antifungal activity (MFC = 32–2 μg/mL) against the *Candida* reference strains and clinical isolates. Considering the structure-dependent effect, we propose that the presence of bromine and chlorine moiety attached to C_2_ of phenyl ring in compound **4** may be the reason of its high antifungal activity (Bondaryk et al., 2015). As we showed previously, these elements are important for the interactions with fungal cells (Bondaryk et al., 2015; Staniszewska et al., 2015a,b). Indeed, bromodichloro- and dichloro-methyl substituents have a crucial role for reducing morphogenesis and adhesion of *C. albicans* without enhanced toxicity. We studied the antifungal activity of new compound **4** against a variety of clinical isolates, including *Candida* species that are difficult to treat, such as *C. glabrata, C. krusei, C. parapsilosis*, and *C. tropicalis*. All strains of *Candida* were classified as susceptible to new compound **4**. Since, nearly 20% of *C. glabrata* strains exhibit intrinsic resistance to azoles and its resistance to echinocandins is on the rise (with >10% of isolates showing resistance; Shor and Perlin, 2015), we showed that compound **4** can be an alternative against these multi-drug resistant strains. A high mortality rate, in patients infected with *C. glabrata* (80%) or *C. tropicalis* (77%; Bonfietti et al., 2012), indicates the need to search for new, effective antifungals against candidiasis. We found an epidemiological relationship between the cases affected by *Candida* isolates and the sensitivity to compound **4** (Tables 2, 3).

As PG is more common among *Candida* strains and stems from compensatory responses to the inhibition of glucan synthesis, our data strongly imply that compound **4** is unlikely impact this effect in clinical strains. PG was evident exclusively for only one clinical isolate, respectively, from species of *C. glabrata* and *C. parapsilosis* and reference strain 14053 (3 out of 63 strains). This is not in line with previous studies (Chamilos et al., 2007), where PG was described mainly for *C. krusei* and *C. tropicalis*. To our knowledge, Shields et al. (2011) reported paradoxical growth of *C. glabrata*. Despite findings of Chamilos et al. (2007), it ought to be indicated that it is species-related. PG has been demonstrated previously by other authors (Bizerra et al., 2011; Shields et al., 2011; Rueda et al., 2014) using a broth microdilution assay for detecting MIC and MFC mainly for echinocandins. In our study, the MFC assay was helpful in identifying PG of clinical isolates in the presence of concentrations that exceeded MICs of compound **4** (2–16 μg/mL), especially for *C. glabrata* and *C. parapsilosis*. In fact, the MFC assay revealed PG that was not evident by broth microdilution. To our knowledge, this is the first study to demonstrate that compound **4** was associated with its own PG when it was tested in the presence of concentration ranging from 2 to 16 μg/mL. Our findings were consistent with earlier reports suggested that this effect represents generalized compensatory responses to a range of cell wall insults, rather than specific responses to glucan synthesis. We showed that the MIC values of compound **4** were unaltered in the presence of osmotic protector (sorbitol), suggesting compound **4** does not act by inhibiting fungal cell wall synthesis.

In the study, a biphasic killing of *C. albicans* biofilms was observed, with the small population being killed at relatively low compound **4** concentrations, and abundant fraction of cells (persistent cells), remaining resistant at high concentration of agent. Moreover, structural analyses revealed that compound **4**-treated biofilms showed minor defects in the biofilm architecture (Figure 7). In our opinion, the reintroduction of fluorine atom instead of chlorine at the C_4_ position of the benzene ring can exert activity against *Candida* sessile cells (Bondaryk et al., 2015; Staniszewska et al., 2015a,b). *Candida albicans* biofilm heterogeneity has a direct impact on antifungal sensitivity to compound **4**, based on our *in vitro* study. Our data illustrate significant differences between planktonic and sessile cells of *C. albicans*. While amphotericin B is equally effective against both growing forms, compound **4** was less effective against mature biofilms. Furthermore, we found a PG of lower percentage metabolic reduction with higher concentration of compound **4** than with 0.5 μg/mL *in vitro* (Figure 7). The exact mechanism causing this PG with *Candida* strains and its clinical relevance are unknown.

Following validation of antifungal activity, compound **4** was finally checked for its toxicity, in order to provide evidence for future clinical use. Overall, no cytotoxic activity was detected against mammalian cells *in vitro* as well as *G. mellonella in vivo*.

Finally, our findings with compound **4** suggest a general strategy for antifungal agent development that might be useful in limiting the emergence of resistance in *Candida* strains. As caspofungin and amphotericin B exerted selective pressure on *C. glabrata*, compound **4** can be alternative in patients at risk for bloodstream infection. Characterization of various phenotypes of clinical strains in the presence of compound **4** would indicate that this antifungal agent is likely to impact the treatment of patients with candidiasis. Our findings support the view that β-ketosulfone warrant further explanation of their biological properties and toxicity in non-clinical studies and clinical trials.

## Author contributions

MS: substantial contribution to conception and design of the whole manuscript (know-how), contribution in all experiments included in the manuscript, and acquisition of data, and analysis and interpretation of data, revising it critically for important intellectual content; MB: participates in the experimental part: RT-PCR, adhesion study, cytotoxicity and biofilm studies. MW: contribution in the experimental part: cytotoxicity on Vero cells EE: contribution in experimental part refers to antifungal activity of compound **4**. HM: participates in revising the manuscript critically for important intellectual content. ZO: contribution in acquisition of data on synthesis of beta-ketosulfone, substantial contributions to conception and design of sulfone chemical synthesis.

### Conflict of interest statement

The authors declare that the research was conducted in the absence of any commercial or financial relationships that could be construed as a potential conflict of interest.
